# Rituximab for antibody-negative combined central and peripheral demyelination presenting with acute respiratory failure: a case report and literature review

**DOI:** 10.3389/fimmu.2025.1682211

**Published:** 2025-10-17

**Authors:** Yuanjiang Zheng, Jiangyan Hou, Youjun Jiang, Jianglin Yu, Ni Zhang, Xianwei Ye

**Affiliations:** ^1^ Department of Respiratory and Critical Care Medicine, Guizhou Provincial People’s Hospital, Guiyang, Guizhou, China; ^2^ Graduate School, Zunyi Medical University, Zunyi, Guizhou, China; ^3^ Department of Neurology, Guizhou Provincial People’s Hospital, Guiyang, Guizhou, China

**Keywords:** combined central and peripheral demyelination, respiratory failure, B cells, IL-6, rituximab, immunotherapy

## Abstract

Antibody-negative combined central and peripheral demyelination (CCPD) is a rare neuroimmunological disorder with heterogeneous manifestations and variable treatment response. We report a case of a 40-year-old man who developed acute hypercapnic respiratory failure following influenza A infection. Neuroimaging revealed demyelinating lesions in the brainstem and spinal cord, and nerve conduction studies showed multifocal peripheral demyelination. Peripheral immune profiling demonstrated marked B-cell activation and elevated serum interleukin-6 (IL-6). The patient responded poorly to corticosteroids, intravenous immunoglobulin, and plasma exchange, but improved rapidly after two consecutive daily doses of 100 mg rituximab (RTX), which was accompanied by a significant reduction in IL-6 levels, CD19^+^ B-cell depletion, ventilator weaning, and marked neurological recovery. This case underscores the importance of considering neuroimmunological causes in acute hypercapnic respiratory failure of unclear etiology. The close temporal association between IL-6 decline, B-cell depletion, and clinical improvement suggests that the B-cell–IL-6 axis may play a central role in antibody-negative CCPD pathogenesis. Low-dose RTX may represent a safe and effective therapeutic option in refractory cases.

## 1 Introduction

CCPD is a rare but clinically heterogeneous autoimmune neurological disorder with poorly understood pathogenesis and immunological characteristics (1). Studies have shown that approximately 65% of patients report preceding upper respiratory or gastrointestinal infections (2), suggesting that a “molecular mimicry” mechanism—via activation of autoreactive T and B cells and subsequent cross−reactive immune responses against myelin antigens—may be a key pathogenic trigger. In some cases, autoantibodies such as anti−AQP4, MOG, or NF155 have been detected, supporting an antibody−mediated mechanism. However, many patients remain seronegative and show poor response to standard immunotherapies, indicating the possible involvement of antibody−independent immune pathways (3).

B cells play multifaceted roles beyond antibody production. They participate in antigen presentation and collaborate with CD4^+^ T cells to activate the Th1/Th17 axis, thereby amplifying local inflammatory responses. These pro−inflammatory effects become more pronounced when the blood–brain barrier (BBB) is compromised (4). In recent years, growing attention has been given to the role of B−cell–derived IL−6 in demyelinating pathologies. IL−6 promotes the differentiation of Th17 cells and disrupts BBB integrity via the JAK/STAT3 signaling pathway, thereby intensifying central nervous system inflammation. These mechanisms have been well established in multiple sclerosis (MS) and neuromyelitis optica spectrum disorder (NMOSD) (4, 5). Given the limited efficacy of conventional immunotherapies in some CCPD patients, RTX, a B−cell–targeted agent, has emerged as a novel therapeutic approach whose role in CCPD warrants further investigation (6, 7).

In this report, we describe a case of antibody−negative CCPD initially presenting with respiratory failure. Dynamic profiling of peripheral immune phenotypes and cytokine levels revealed that B−cell activation and elevated IL−6 were closely associated with disease activity. Following RTX treatment, the patient exhibited marked clinical improvement. These findings suggest that the B−cell–IL−6 axis may play a central pathogenic role in antibody−negative CCPD and offer valuable insights into its underlying mechanisms and potential avenues for precision therapy.

## 2 Case presentation

**Figure 1:**
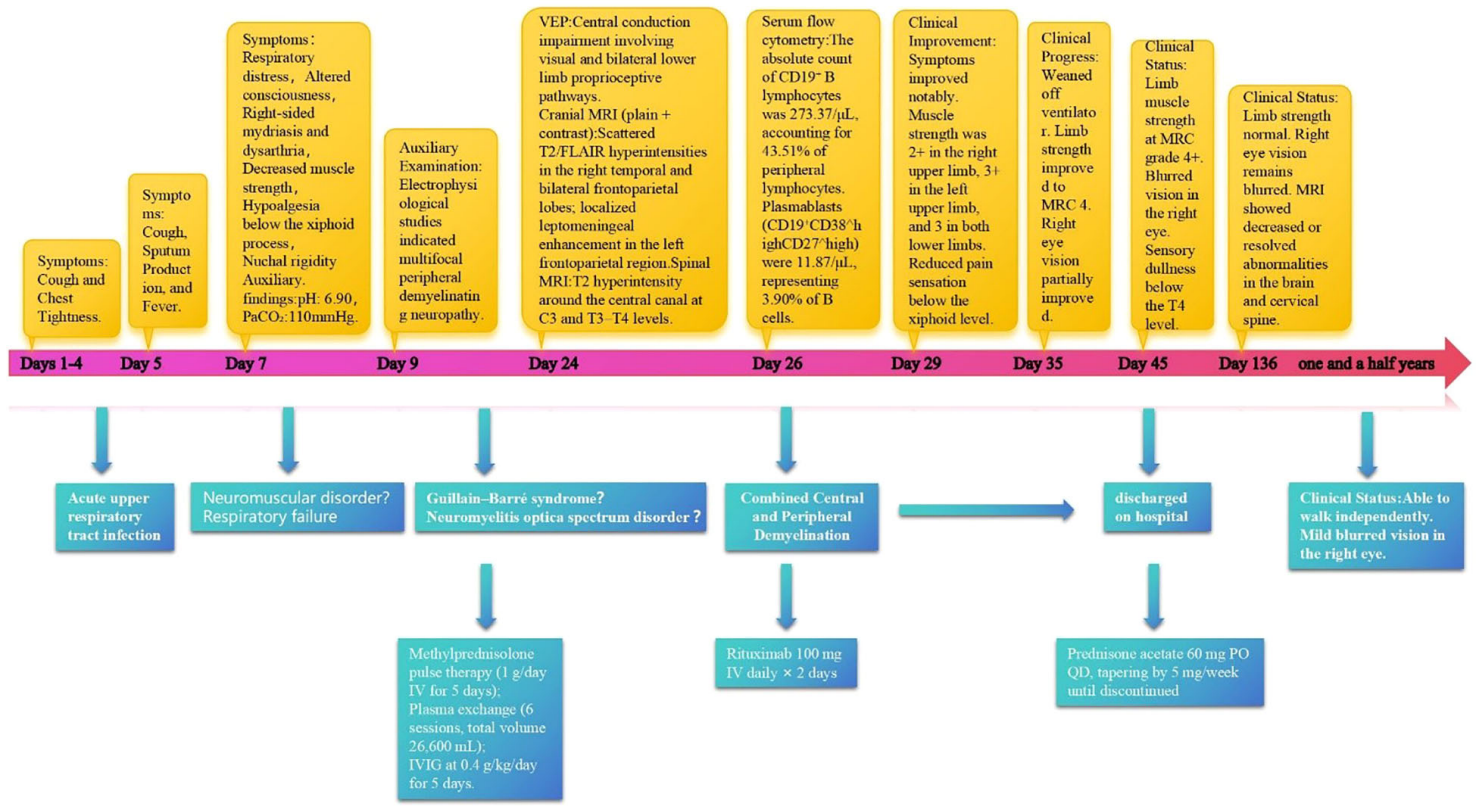
Diagnostic and therapeutic timeline of a patient with combined central and peripheral demyelination.

**Figure 2:**
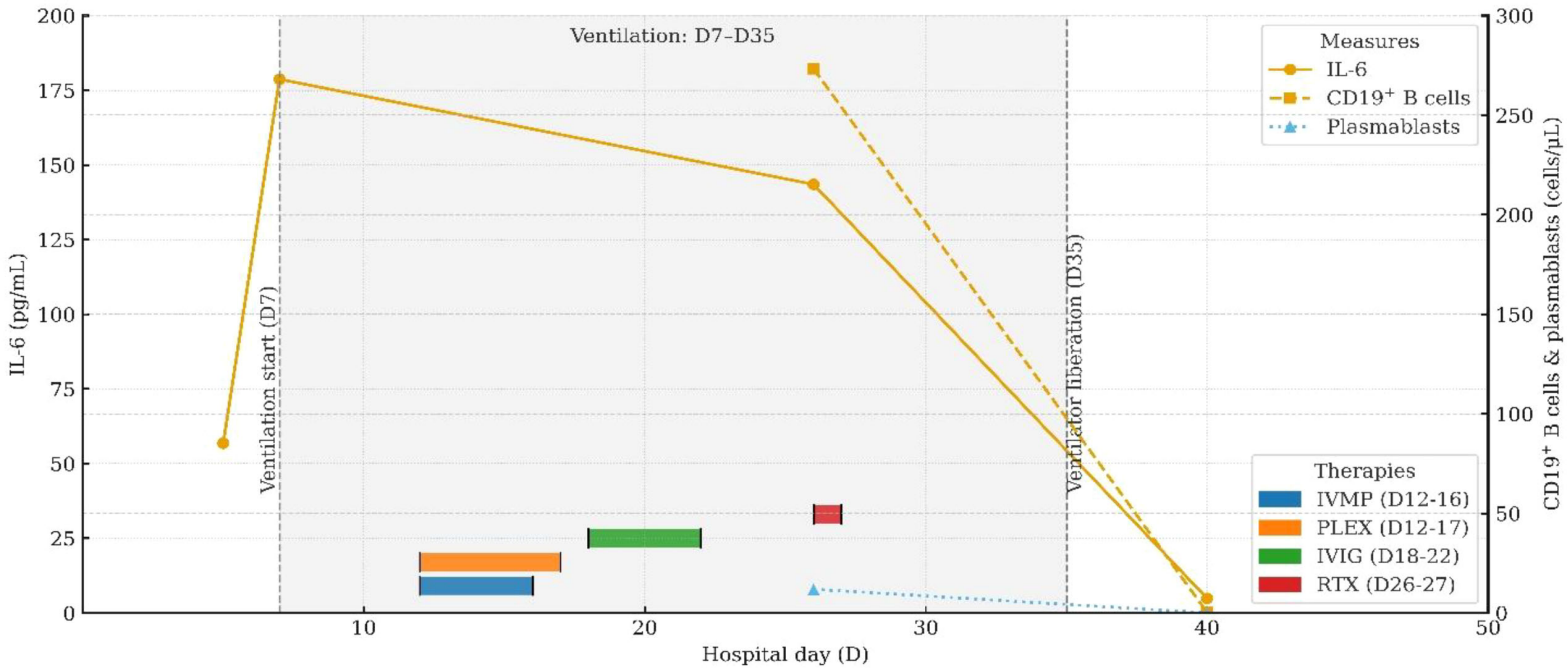
Longitudinal IL-6 and B-cell (plasmablast) dynamics aligned with therapy milestones and ventilatory course. Detailed data are provided in **Supplementary Table S1**.

This case report documents the patient’s clinical course from day 1 of symptom onset through post-treatment follow-up, presenting a timeline of symptom evolution, therapeutic interventions, and long-term management (**Figure 1**, **Supplementary Table S1**). The patient was a previously healthy 40-year-old man with no history of smoking, alcohol use, or comorbid conditions. During the initial phase (days 1–4), he experienced dry cough and chest tightness without sputum production; oral antiviral agents and antitussives provided limited benefit. On day 5, productive cough and fever worsened (maximum 38.6 °C) without overt dyspnea. Upon admission, vital signs were overall stable. A nucleic acid amplification test on an oropharyngeal swab was positive for influenza A virus. Routine laboratory tests indicated a nonspecific inflammatory response, with serum IL-6 of 56.8 pg/mL (**Supplementary Table S2**, **Figure 2**). Routine antibody screening was negative (**Supplementary Table S3**). Chest computed tomography showed no abnormalities, and neurological examination revealed no focal deficits.

**Figure 3:**
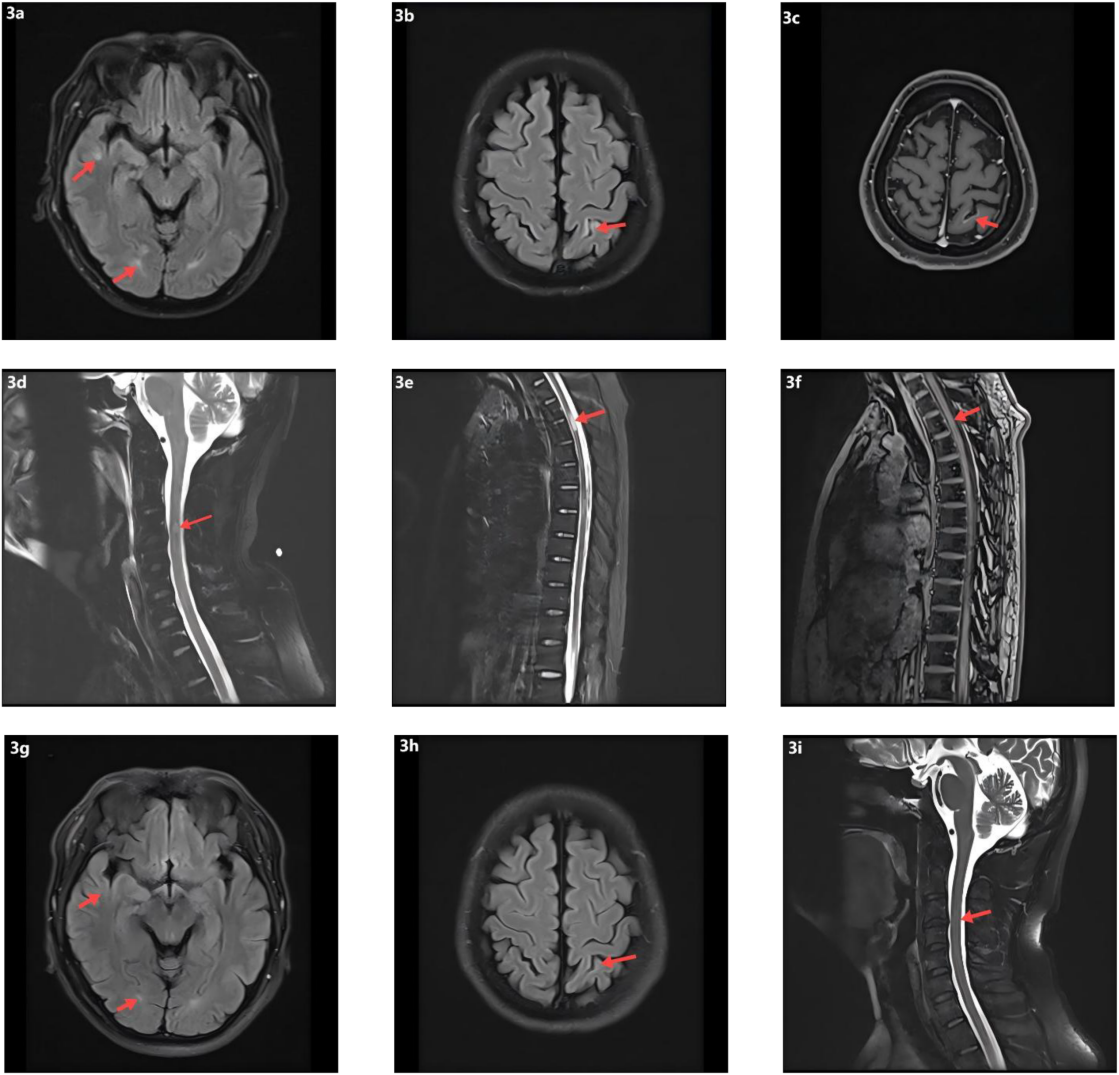
Neuroimaging findings and follow-up MRI. **(a)** Scattered punctate T2-weighted and FLAIR hyperintense lesions in the right temporal and frontoparietal lobes. **(b)** Similar scattered hyperintense lesions in the left frontoparietal cortex on T2-weighted and FLAIR sequences. **(c)** Post-contrast T1-weighted imaging shows focal leptomeningeal enhancement in the left frontoparietal region. **(d)** T2 hyperintensity surrounding the central canal at the C3 level of the cervical spinal cord. **(e)** T2 hyperintensity around the central canal at the T3–T4 levels of the thoracic spinal cord. **(f)** Mild contrast enhancement of the T3–T4 spinal cord lesions on post-contrast T1-weighted images. **(g)** Follow-up T2-FLAIR images demonstrate resolution of the hyperintense lesions in the right temporal and frontoparietal lobes. **(h)** Decreased FLAIR hyperintensity in the left parietal lobe on follow-up imaging. **(i)** Resolution of previously abnormal T2 signal in the cervical spinal cord.

On day 7, the patient abruptly developed severe ventilatory failure (arterial blood gas: pH 6.90, PaCO_2_ 115 mmHg, PaO_2_ 56 mmHg) and was transferred to the intensive care unit for endotracheal intubation and mechanical ventilation. Concurrently, right-sided mydriasis; dysarthria/dysphagia; limb weakness [Medical Research Council (MRC) grades: right upper 2/5, left upper 3/5, both lower limbs 2/5]; absence of deep tendon reflexes in all limbs; negative Babinski and Kernig signs bilaterally; reduced sensation below the T4 level; and nuchal rigidity emerged. Lumbar puncture demonstrated albuminocytologic dissociation (**Supplementary Table S4**); serum IL-6 was markedly elevated at 178.7 pg/mL; and chest CT showed minimal pulmonary exudative changes. Comprehensive neuroimmunological evaluation demonstrated that Cerebrospinal fluid (CSF) oligoclonal bands were negative; assays for central and peripheral nervous system–related autoantibodies (anti-GQ1b, anti-NF155, anti-AQP4, anti-MOG, anti-MAG; see **Supplementary Table S3**) were all negative. CSF infectious testing—including metagenomic next-generation sequencing (mNGS)—did not identify a specific pathogen (**Supplementary Table S4**). On day 9, electrodiagnostic testing revealed multifocal demyelinating changes of the peripheral nerves (**Supplementary Table S5**), and the initial differential diagnosis included GBS versus NMOSD. Beginning on day 12, high-dose intravenous methylprednisolone (1 g/day for 5 consecutive days) was administered in combination with therapeutic plasma exchange (six sessions; total exchange volume 26 600 mL); however, there was no improvement in neurological or respiratory status (muscle strength unchanged with persistent sensory deficits). On day 16, repeat lumbar puncture showed the opening pressure had decreased to 220 mmH_2_O, while protein had increased to 0.67 g/L (**Supplementary Table S4**); the autoimmune encephalitis antibody panel remained negative (**Supplementary Table S3**). On day 18, intravenous immunoglobulin (IVIG, 0.4 g/kg/day for 5 days) was given, but neurological and respiratory function still did not improve. On day 24, visual evoked potentials indicated central conduction impairment (**Supplementary Table S6**); brain MRI demonstrated scattered cortical T2/FLAIR hyperintensities in the right temporal and bilateral frontoparietal regions with mild leptomeningeal enhancement in the left frontoparietal area (**Figures 3a–c**), while cervical/thoracic spinal MRI showed central canal–adjacent T2 hyperintensities at C3 and T3–T4 with mild enhancement (**Figures 3d–f**). Integrating the imaging and neurophysiological findings with the clinical phenotype and prior literature (2, 3, 7), a final diagnosis of CCPD was established.

**Figure 4:**
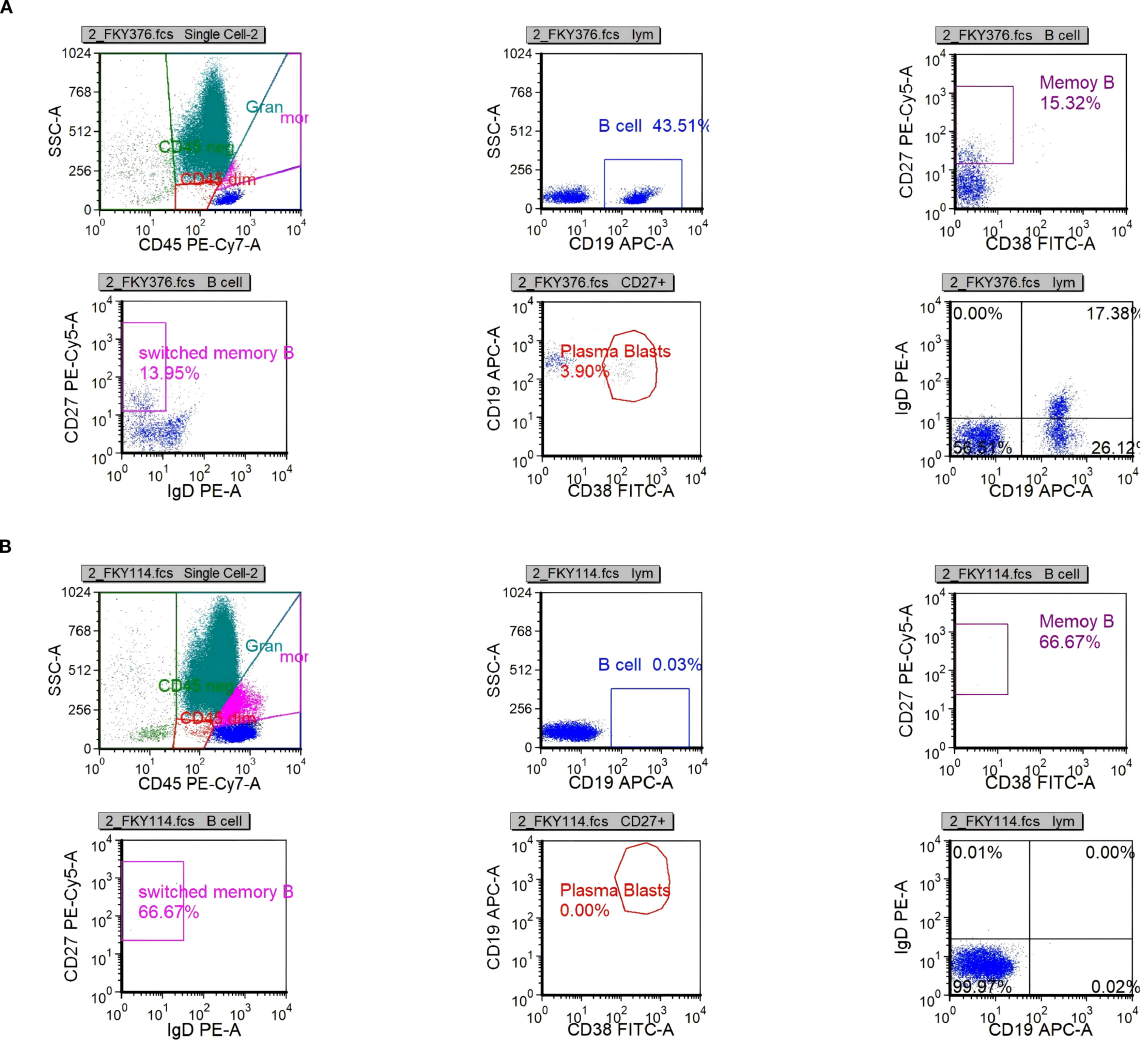
Scatter plots of B-cell analysis by flow cytometry. **(a)** B-cell population (CD19^+^) on day 26. **(b)** B-cell population (CD19^+^) on day 40.

On day 26, immunologic evaluation of peripheral blood demonstrated expansion of CD19^+^ B cells to 273.37 cells/μL (43.51% of lymphocytes) and plasmablasts to 11.87 cells/μL (3.90% of B cells) (**Figure 4a**, **Supplementary Table S7**); meanwhile, IL-6 remained at 143.5 pg/mL, with only a modest decline from the previous level. The remaining lymphocyte subsets and the CD4^+^/CD8^+^ ratio were within normal limits (**Supplementary Table S3**), indicating a B-cell–driven inflammatory response. Accordingly, RTX was administered at 100 mg/day for 2 days. Pre-RTX screening for HBV/TB and other blood-borne infections was negative, and the infusions were delivered in the ICU with standard premedication and continuous monitoring without adverse reactions. A By day 29, a rapid clinical and immunologic response was evident, with clear improvement in muscle strength (right upper limb 2+, left upper limb 3+, both lower limbs 3), restoration of deep tendon reflexes in both lower limbs, and resolution of nuchal rigidity. By day 35, the patient was successfully weaned from mechanical ventilation, PaCO_2_ decreased to 48 mmHg, limb strength reached grade 4, and vision in the right eye improved. By day 40, CD19^+^ cells had declined to 0.25 cells/μL, plasmablasts were undetectable (**Figure 4b**, **Supplementary Table S8**), and IL-6 had fallen to 4.85 pg/mL. Given the significant clinical and immunological response, no further doses of RTX were administered.

At the time of discharge on day 45, the patient had regained muscle strength to MRC grade 4+ in all four limbs, with deep tendon reflexes normalized. However, diminished sensation below the T4 level and mild blurred vision in the right eye persisted. Oral prednisone acetate (60 mg/day) was prescribed at discharge and tapered by 5 mg per week until discontinuation. At the follow-up visit on day 136, muscle strength had fully recovered, although mild visual blurring in the right eye remained. Repeat MRI showed near-complete resolution of the brain parenchymal lesions and complete disappearance of abnormal cervical spinal cord signals (**Figures 3g–i**). The patient declined further neurophysiological testing. At the 18-month follow-up, the only residual symptom was mild blurring of vision in the right eye, and no relapses had occurred. In view of the rapid and durable remission achieved with low-dose RTX, we adopted an observation-first management strategy without scheduled maintenance therapy; re-treatment would be triggered by CD19^+^ B-cell reconstitution—monitored by flow cytometry when feasible—or by new clinical or MRI evidence of disease activity. Notably, the patient declined flow-cytometric monitoring during follow-up.

### 2.1 Methods: immunological assays

#### 2.1.1 Serum IL-6 quantification

Serum was prepared per standard procedures; when same-day analysis was not feasible, aliquots were stored at −80 °C with no more than one freeze–thaw cycle. IL-6 was measured by electrochemiluminescence immunoassay (ECLIA; Elecsys IL-6, Roche Diagnostics) on the cobas e platform. The analytical measuring range was 1.5–5 000 pg/mL with a limit of detection of 1.5 pg/mL; specimens exceeding the upper limit were re-assayed after 1:2 dilution. All samples were tested in duplicate, with two-level daily internal quality controls applied in accordance with Westgard rules. The intra-assay coefficient of variation (CV) was ≤5% and the inter-assay CV was ≤8%.

#### 2.1.2 Peripheral B-cell immunophenotyping

EDTA-anticoagulated whole blood was processed within 6 hours of collection. Acquisition was performed on a BD FACSCanto II flow cytometer with daily Cytometer Setup & Tracking (CS&T) bead calibration. The antibody panel comprised CD45, CD3, CD19, CD27, and CD38 (all from BD Biosciences). Compensation and fluorescence-minus-one (FMO) controls were included. Lymphocytes were identified by FSC/SSC characteristics and CD45^bright expression, with singlets retained by FSC-A/FSC-H gating. B cells were defined as CD19^+^, and plasmablasts as CD19^+^CD38^highCD27^high. Absolute counts were obtained using a single-platform bead-based method (BD TruCOUNT) and reported as cells/μL; subset frequencies were expressed as a percentage of total lymphocytes or of CD19^+^ B cells, as appropriate. Reproducibility assessments showed CV <4% for %CD19^+^, CV <10% for plasmablast frequency, and CV <6% for absolute counts. Data were analyzed in FlowJo v10, with inter-operator variability <5%.

## 3 Discussion

This case presents several notable clinical features that merit further discussion. First, the patient had a clearly documented history of upper respiratory tract infection preceding disease onset, raising the possibility of molecular mimicry as a trigger for aberrant autoimmune activation (8). This mechanism—whereby structural similarities between microbial antigens and host proteins lead to misdirected immune responses—has been widely implicated in a variety of immune-mediated neurological disorders (9). Second, the initial presentation with acute respiratory failure is exceedingly rare among reported cases of CCPD. Hypercapnic respiratory failure is typically observed in chronic obstructive pulmonary disease or neuromuscular conditions but may also occur in demyelinating diseases due to simultaneous central and peripheral involvement. Although diaphragmatic ultrasonography to assess diaphragmatic paralysis was not performed at the onset of ventilatory failure, the baseline chest CT at admission was unremarkable. Moreover, an immediate repeat chest CT obtained after the development of respiratory failure showed only minimal exudative changes without the diffuse, bilateral pulmonary infiltrates characteristic of ARDS, arguing against ARDS. Accordingly, the respiratory failure was more likely attributable to neuromuscular involvement. Central lesions, particularly in the brainstem, can disrupt the chemosensory response to rising CO_2_ levels, while peripheral damage to the phrenic and intercostal nerves compromises respiratory muscle strength and reduces maximal inspiratory pressure (10, 11). In this case, the patient developed severe hypercapnia with PaCO_2_ reaching 115 mmHg, reflecting profound ventilatory insufficiency. The underlying pathophysiology likely involved demyelinating lesions in both the brainstem and the spinal cord from C3 to T4, impairing medullary chemoreceptor sensitivity. In parallel, involvement of the phrenic nerves (C3–C5) and intercostal nerves (T1–T12) further weakened the respiratory pump mechanism. The synergistic effects of central ventilatory dysregulation and peripheral neuromuscular dysfunction culminated in life-threatening respiratory failure. In addition, several hereditary disorders can present with CCPD and may overlap with acute immune-mediated demyelinating processes, including Charcot–Marie–Tooth disease (particularly GJB1-related/CMTX1), mitochondrial disorders, and leukodystrophies. Nevertheless, the acute/subacute clinical course in this patient, the characteristic paraclinical findings, and the favorable response to immunotherapy collectively support an immune-mediated pathogenesis. Given these phenotypic overlaps, we recommend that genetic testing be routinely considered—and performed when appropriate—during the diagnostic evaluation of CCPD to further refine the differential diagnosis.

Moreover, the absence of OCBs in the CSF distinguishes this case from classical central demyelinating disorders such as MS and NMOSD, suggesting that CCPD may possess distinct immunopathological characteristics. Although traditional paradigms emphasize the central role of T cells in autoimmune demyelinating diseases (12, 13), the clinical efficacy of B-cell-depleting agents such as RTX in both MS and NMOSD highlights the emerging pathogenic importance of B cells (14, 15). Viral infections are recognized as key triggers of aberrant B-cell activation. They can stimulate antigen-presenting cells to release pro-inflammatory cytokines such as type I interferons, IL-6, and IL-1, thereby amplifying B-cell responses, driving autoantibody production, promoting Th17 polarization, and suppressing protective Th1 immunity—collectively fostering a pro-inflammatory milieu (16). For example, Epstein–Barr virus has been implicated in NMOSD exacerbations through upregulation of EBI2 expression, with EBI2^+^CD19^+^ B cells elevated during acute episodes and associated with serum IL-6 and IL-10 levels (17). Under inflammatory conditions, B cells can secrete high levels of IL-6 and GM-CSF, two cytokines with critical roles in demyelinating pathophysiology. IL-6 contributes to central inflammation via the JAK/STAT3 signaling pathway by disrupting BBB integrity, inducing MMP-9 expression, and increasing vascular permeability (18–20). It also promotes neutrophil infiltration into the CNS, exacerbating the inflammatory response. GM-CSF further augments CNS autoimmunity by enhancing dendritic cell activity and promoting Th1 polarization (21, 22). In the present case, a preceding viral infection was documented. During disease progression, peripheral blood flow cytometry showed normal counts and proportions of CD4^+^ and CD8^+^ T cells, but a marked expansion of B cells and activated plasmablasts, along with significantly elevated serum IL-6 levels. Notably, IL-6 levels dynamically paralleled disease activity and declined rapidly following RTX administration. These observations suggest that IL-6 not only contributes to the amplification of neuroinflammation but may also serve as a potential biomarker of disease activity. Phase-3 evidence from NMOSD provides mechanistic and clinical context: the anti-IL-6 receptor monoclonal antibody satralizumab significantly reduced relapse risk in two randomized trials—SAkuraSky as add-on therapy (overall HR 0.38; AQP4-IgG-positive subgroup HR 0.21) and SAkuraStar as monotherapy (overall HR 0.45)—with favorable safety profiles (23, 24). Moreover, in the randomized TANGO study, tocilizumab outperformed azathioprine in highly relapsing NMOSD (HR 0.236) and prolonged time-to-first relapse (25). In MS, large cohort studies demonstrate that intrathecal IL-6 associates with disability and progressive disease biology, reinforcing its candidacy as a central inflammatory biomarker (26, 27). However, clinical experience with IL-6 pathway blockade in MS remains limited and heterogeneous—ranging from isolated benefit in fulminant pediatric MS to sporadic reports of MS onset during IL-6R inhibition for rheumatoid arthritis (28, 29)—and its therapeutic value in MS requires further evaluation (30). Taken together, our observations and prior data suggest that an immunopathogenic axis centered on B cells and IL-6 may be pivotal in antibody-negative CCPD. Serial IL-6 measurement may reflect neuroinflammatory burden and treatment response and merits prospective validation as a monitoring biomarker in CCPD; in selected refractory cases, IL-6 pathway inhibition could complement B-cell–directed therapy, although its efficacy and safety require further confirmation.

According to previous reports, the rates of clinical improvement in CCPD patients treated with corticosteroids, IVIG, and plasma exchange are approximately 83.3%, 66.7%, and 87.5%, respectively, whereas interferon-β has demonstrated efficacy in only 10% of cases (3). Most patients respond favorably to intravenous corticosteroid therapy; however, for those with suboptimal response, combination strategies may be considered, including IVIG (3, 31, 32), plasma exchange (3, 31, 32), or targeted immunotherapies such as RTX (2), natalizumab (2), or fingolimod (33). Originally developed for the treatment of B-cell lymphomas, RTX has gained broad application in autoimmune demyelinating diseases such as MS, NMOSD, and myelin oligodendrocyte glycoprotein-associated disease. Its therapeutic potential in CCPD has garnered increasing attention. A review of published CCPD cases treated with RTX (34–39) (**Table 1**) underscores its significant immunological and clinical benefits. From an immunopathogenic perspective, CCPD likely results from a complex interplay between multiple autoantibodies and immune effector cells, among which B cells play a pivotal role in antibody production, antigen presentation, and immune modulation. RTX targets CD20^+^ B cells, thereby interrupting key disease-driving mechanisms. Clinically, RTX has shown consistent efficacy across diverse CCPD phenotypes, age groups, and disease stages. It has proven effective in both seropositive patients with anti-NF155 antibodies [e.g., cases reported by Sökmen (38) and Lambrianides (39)] and in seronegative presentations, as in our case. From a dosing perspective, RTX induction regimens in CCPD span low-dose strategies (cumulative dose ≤200 mg) and conventional/high-dose approaches (cumulative dose ≥1 g). Low-dose protocols—exemplified by our patient (100 mg on two consecutive days) and the report by Zhou et al. (100 mg × 2 one week apart)—were associated with early clinical gains despite refractoriness to corticosteroids, IVIG, and plasma exchange. In our case, the clinical inflection point coincided with rapid depletion of CD19^+^ B cells and a decline in serum IL-6, followed by successful liberation from mechanical ventilation and stepwise motor recovery. By contrast, conventional/high-dose regimens (e.g., 1 g × 2 separated by 2 weeks; 375 mg/m² weekly × 4; or 500 mg weekly × 4 in pediatric settings) likewise yielded substantial neurological improvement, resolution of MRI lesions, and, in some instances, reductions in EDSS scores to near-normal levels. Notably, across published CCPD cases, no serious RTX-related adverse events have been reported, supporting a favorable safety profile—even when RTX was co-administered with other immunosuppressants or disease-modifying therapies. Taken together, these observations suggest that short-term benefit may depend more on the kinetics of B-cell depletion and timely control at the inflammatory peak than on cumulative induction dose per se. In antibody-negative CCPD—where pathogenic antibody drivers remain uncertain—linking clinical responses to immune readouts (e.g., CD19^+^ counts, plasmablast levels, and IL-6 trajectories) may help calibrate RTX induction intensity and maintenance intervals. Moreover, among cases with available follow-up (4–28 months), no relapses were documented after RTX, indicating potential for disease control within this time frame. In conclusion, RTX appears to offer multiple therapeutic advantages in CCPD, including a clearly defined mechanism of action, broad clinical efficacy, applicability across heterogeneous patient populations, and good safety and tolerability. Although large-scale controlled trials are still lacking, accumulating clinical evidence supports its role as a promising treatment option, particularly in refractory or relapsing CCPD.

**Table 1:** Summary of RTX-treated CCPD cases in published literature.

Paper	Patient ID	Age(years)	Gender	Clinical features	CSF findings	Conventional immunotherapy	RTX regimen	Outcomes	Follow-up (months)	Relapse status after RTX
Savasta et al. (34)	1	14	M	Refractory CCPD with multiple relapses	Oligoclonal bands(−),anti-GQ1b/NF155/AQP4/MOG (−)	Corticosteroids, IVIG, Natalizumab.	500 mg/week × 4 weeks	EDSS improved from 7 to 1; MRI lesions resolved	28	No relapse
Zhou et al. (35).	2	14	F	Acute myelitis progressing to GBS with HEV infection	Oligoclonal bands(−),anti-AQP4/MOG/ganglioside (−)	Corticosteroids, IVIG, PLEX	100 mg × 2 doses (1-week interval)	Motor recovery (upper limbs: 5/5, lower limbs: 3/5); spinal lesions improved	4	No relapse
Farag et al. (36).	3	46	M	IDP with CNS demyelination, familial MS history	Oligoclonal bands(+),anti-AQP4/MOG (−)	Corticosteroids, IVIG, PLEX	1 g × 2 doses (2-week interval)	Clinical and radiological improvement	NR	NR
Makkawi et al (37).	4	29	M	Acute quadriplegia with respiratory failure	Oligoclonal bands(−),anti-NF155/AQP4/MOG (−)	Corticosteroids, IVIG, Azathioprine	1 g × 2 doses (2-week interval)	Full motor recovery	12	No relapse reported (EDSS 2.5 at 1 year)
Sokmen et al. (38).	5	18	F	NF-155 IgG(+) CCPD, IVIg-resistant	Anti-NF-155 (+),oligoclonal bands (−)	Corticosteroids, IVIG	375 mg/m² weekly × 4 weeks	Motor recovery with residual sensory deficits	NR	NR
Lambrianides et al. (39).	6	30	M	NF-155(+) CCPD, familial MS history, testicular cancer	Anti-NF-155 (+), elevated CSF protein	None	1 g × 2 doses (2-week interval)	Stable condition, partial symptom relief	NR	NR
Our case	7	40	M	Type II respiratory failure as initial presentation	Oligoclonal bands(−), anti-GQ1b/ NF155/AQP4/MOG (−)	Corticosteroids, PLEX, IVIG	100 mg × 2 days	Successful extubation, mRS 1, residual visual impairment.	18	No relapse

CCPD, combined central and peripheral demyelination; IVIG, intravenous immunoglobulin; PLEX, plasma exchange; EDSS, Expanded Disability Status Scale; mRS, Modified Rankin Scale; NR, not reported.

## 4 Conclusion

This case underscores that CCPD, although rare, can initially manifest as acute hypercapnic respiratory failure—a presentation frequently misattributed to primary pulmonary disorders. Such atypical clinical manifestations emphasize the importance of heightened diagnostic vigilance and the consideration of underlying neuroimmunological etiologies in patients with otherwise unexplained ventilatory dysfunction. Longitudinal monitoring in our case revealed that serum IL-6 levels closely paralleled disease activity, suggesting that IL-6 may serve as a potential biomarker for inflammatory activity in antibody-negative CCPD. Remarkably, the patient demonstrated rapid neurological recovery and a substantial reduction in IL-6 levels following low-dose RTX therapy, despite exhibiting poor responsiveness to conventional immunomodulatory treatments. These findings indicate that RTX may represent a promising and well-tolerated therapeutic option for antibody-negative CCPD, meriting further systematic evaluation. Moreover, this case provides preliminary evidence supporting a pathogenic role for the B-cell–IL-6 axis in antibody-negative CCPD involving both central and peripheral demyelination. However, causality between the decline in IL-6 and clinical improvement cannot be established in a single case report; available antibody panels may not capture all relevant antigenic targets, and subclinical relapses cannot be excluded. Future mechanistic and controlled studies are needed to clarify this axis and to define optimal patient selection, dosing, and monitoring strategies.

## 5 Patient perspective

The patient reported intense fear and loss of control at the onset of hypercapnic respiratory failure and described the period of limited response to corticosteroids, plasma exchange, and IVIG as “discouraging.” Following RTX, he perceived a rapid and meaningful change in breathing effort, voice strength, and mobility, with ventilator weaning described as “the moment I felt I was getting my life back.” He emphasized the value of frequent communication with the care team and expressed willingness for his anonymized story to support others with similar disorders.

## Data Availability

The original contributions presented in the study are included in the article/**Supplementary Material**. Further inquiries can be directed to the corresponding author.
