# Supplementary material for: Rituximab for antibody-negative combined central and peripheral demyelination presenting with acute respiratory failure: a case report and literature review
Source: Front Immunol. 2025 Oct 17;16:1682211. doi: 10.3389/fimmu.2025.1682211 (PMC12575319; doi:10.3389/fimmu.2025.1682211)
Supplement: Supplementary file 1 [file DataSheet1.pdf]

Supplementary Material

1 Supplementary Data

Table1 . Integrated Timeline Aligning Laboratory/Immunologic Markers with Clinical Events and Therapeutic Interventions

Day	Key labs	Interventions (dose, duration)	Neurological status	Imaging / Neurophysiology	Clinical response
1–6	Routine laboratory testing indicated systemic inflammation without disease-specific abnormalities (Supplementary Table 2); notably, the serum interleukin-6 (IL-6) concentration was markedly elevated at 56.8 pg/mL.	Supportive care	No focal deficits	—	Stable
7	ABG: pH 6.90, $PaCO_2$ 115 mmHg, PaO_2 56 mmHg; CSF albuminocytologic dissociation;Serum IL-6 was markedly elevated at 178.7 pg/mL.	Intubation; mechanical ventilation	Right mydriasis; bulbar symptoms; paraparesis (MRC 2–3/5); T4 sensory level; neck stiffness	MRI ordered (later multifocal demyelination); NCS planned	Stabilized after intubation
12–16	—	IV methylprednisolone 1 g/day ×5	No meaningful improvement	—	Refractory
12–17	—	Therapeutic plasma exchange ×6 sessions	Persistent weakness; ventilator dependence	—	No meaningful response
18–22	—	IVIG 0.4 g/kg/day ×5	No clear improvement	—	Refractory
26	Peripheral $CD19^+$ B cells to 273.37 cells/μL and plasmablasts to 11.87 cells/μL;IL-6 143.5 pg/mL;	Rituximab 100 mg (dose 1 of 2)	Severe limb weakness; ventilator dependence	Multifocal demyelinating lesions (brain/cervical-thoracic cord)	—
27	—	Rituximab 100 mg (dose 2 of 2)	—	—	—

29	—	—	Clear limb strength improvement	—	Improving
35	ABG: $PaCO_2$ 48 mmHg	Liberated from mechanical ventilation	Further strength gains; improved bulbar function	—	Weaned; respiratory improvement
40	$CD19^+$ cells had declined to 0.25 cells/μL, plasmablasts were undetectable; IL-6 4.85 pg/mL	—	Sustained improvement	—	Clinical recovery aligns with immunologic change
45 (discharge)	—	Prednisone taper	MRC 4+; mild residual T4 sensory change; blurred right vision	—	Discharged, no relapse during admission
136 (follow-up MRI)	—	—	Ambulatory	MRI: near-complete lesion resolution	Radiologic improvement

Abbreviations: ABG, arterial blood gas; IVIG, intravenous immunoglobulin; NCS, nerve conduction studies.

Table2. Blood Test Results

Parameter (Unit)	Day 5	Day7	Day 26	Day 40	Day 45	Reference Range
WBC count ($\times 10^9$/L)	15.01	19.10	12.68	11.20	8.88	3.5-9.5
Hemoglobin (Hb) (g/L)	147	132	106	102.0	128	139-184
RBC count ($\times 10^{12}$/L)	4.83	4.35	3.50	3.26	4.23	4.5-6.5
C-reactive protein (mg/L)	61.50	98.30	22.10	9.5	2.30	0-5
Interleukin- 6（pg/mL）	56.8	178.7	143.5	4.85	3.50	0-7
PaO_2 (mmHg)	85	56	97	95		80-100
$PaCO_2$ (mmHg)	36	115	53	37		35-45
$CD19^+$ B Cells (cells/μL）			273.37	0.25		51.00-728.00
Plasmablasts (cells/μL)			11.87	0		0.00-6.00

Table 3. Summary of autoantibodies related to central and peripheral demyelination and infection-related immunologic testing

Cerebrospinal Fluid (CSF) Testing Panel for Autoimmune Peripheral Neuropathy (24 Items)			
No.	Autoantibody Name	Method	Result / Reference Range
1	Anti-Sulfatide Antibody IgG	Immunoblotting	Negative (–) / Negative
2	Anti-GM1 Antibody IgG	Immunoblotting	Negative (–) / Negative
3	Anti-GM2 Antibody IgG	Immunoblotting	Negative (–) / Negative
4	Anti-GM3 Antibody IgG	Immunoblotting	Negative (–) / Negative
5	Anti-GM4 Antibody IgG	Immunoblotting	Negative (–) / Negative
6	Anti-GD1a Antibody IgG	Immunoblotting	Negative (–) / Negative
7	Anti-GD1b Antibody IgG	Immunoblotting	Negative (–) / Negative
8	Anti-GD2 Antibody IgG	Immunoblotting	Negative (–) / Negative
9	Anti-GD3 Antibody IgG	Immunoblotting	Negative (–) / Negative
10	Anti-GT1a Antibody IgG	Immunoblotting	Negative (–) / Negative
11	Anti-GT1b Antibody IgG	Immunoblotting	Negative (–) / Negative
12	Anti-GQ1b Antibody IgG	Immunoblotting	Negative (–) / Negative
13	Anti-Sulfatide Antibody IgM	Immunoblotting	Negative (–) / Negative
14	Anti-GM1 Antibody IgM	Immunoblotting	Negative (–) / Negative
15	Anti-GM2 Antibody IgM	Immunoblotting	Negative (–) / Negative
16	Anti-GM3 Antibody IgM	Immunoblotting	Negative (–) / Negative
17	Anti-GM4 Antibody IgM	Immunoblotting	Negative (–) / Negative
18	Anti-GD1a Antibody IgM	Immunoblotting	Negative (–) / Negative
19	Anti-GD1b Antibody IgM	Immunoblotting	Negative (–) / Negative
20	Anti-GD2 Antibody IgM	Immunoblotting	Negative (–) / Negative
21	Anti-GD3 Antibody IgM	Immunoblotting	Negative (–) / Negative
22	Anti-GT1a Antibody IgM	Immunoblotting	Negative (–) / Negative

23	Anti-GT1b Antibody IgM	Immunoblotting	Negative (–) / Negative
24	Anti-GQ1b Antibody IgM	Immunoblotting	Negative (–) / Negative

Cerebrospinal Fluid (CSF) Testing Panel for Central Nervous System Demyelinating Antibodies(4 Items)

No.	Autoantibody Name	Method	Result / Reference Range
1	Anti-Aquaporin-4 (AQP4) Antibody	Cell-Based Assay (CBA)	Negative (–) / Negative
2	Anti-Myelin Oligodendrocyte Glycoprotein (MOG) Antibody	Cell-Based Assay (CBA)	Negative (–) / Negative
3	Anti-Glial Fibrillary Acidic Protein (GFAP) Antibody	Cell-Based Assay (CBA)	Negative (–) / Negative
4	Anti-Myelin Basic Protein (MBP) Antibody	Cell-Based Assay (CBA)	Negative (–) / Negative

CSF and Serum IgG Levels with Oligoclonal Band (OCB) Analysis(3 Items)

Parameter	Result	Unit	Reference Range	Interpretation
CSF Immunoglobulin G (IgG)	49.6	mg/L	10.0–30.0	Elevated
Serum Immunoglobulin G (IgG)	6.07	g/L	7.0–16.0	Decreased
Oligoclonal Band (OCB) Typing	Negative (–)	—	—	No OCBs in either CSF or serum

Serum Autoantibody Panel for Differential Diagnosis of Autoimmune Encephalitis (15 Items)

No.	Autoantibody Name	Method	Result	Reference Range
1	Anti-Leucine-rich Glioma-Inactivated 1 (LGI1) Antibody IgG	Cell-Based Assay (CBA)	Negative (–)	Negative
2	Anti-Contactin-associated Protein-like 2 (CASPR2) Antibody IgG	Cell-Based Assay (CBA)	Negative (–)	Negative
3	Anti-N-methyl-D-aspartate Receptor (NMDAR) Antibody IgG	Cell-Based Assay (CBA)	Negative (–)	Negative
4	Anti-AMPAR1 Antibody IgG	Cell-Based Assay (CBA)	Negative (–)	Negative

5	Anti-AMPAR2 Antibody IgG	Cell-Based Assay (CBA)	Negative (–)	Negative
6	Anti-GABAB Receptor Antibody IgG	Cell-Based Assay (CBA)	Negative (–)	Negative
7	Anti-GABAA Receptor Antibody	Cell-Based Assay (CBA)	Negative (–)	Negative
8	Anti-mGluR1 Antibody IgG	Cell-Based Assay (CBA)	Negative (–)	Negative
9	Anti-mGluR5 Antibody IgG	Cell-Based Assay (CBA)	Negative (–)	Negative
10	Anti-DPPX Antibody IgG	Cell-Based Assay (CBA)	Negative (–)	Negative
11	Anti-GAD65 Antibody IgG	Cell-Based Assay (CBA)	Negative (–)	Negative
12	Anti-Myelin Oligodendrocyte Glycoprotein (MOG) Antibody	Cell-Based Assay (CBA)	Negative (–)	Negative
13	Anti-Glial Fibrillary Acidic Protein (GFAP) Antibody	Cell-Based Assay (CBA)	Negative (–)	Negative
14	Anti-Aquaporin-4 (AQP4) Antibody	Cell-Based Assay (CBA)	Negative (–)	Negative
15	Anti-mGluR5 Antibody IgG (duplicate)	Cell-Based Assay (CBA)	Negative (–)	Negative
**Serum Autoantibody Panel for Node/Paranode-Related Proteins (5 Items)**				
No.	Autoantibody Name	Method	Result	Reference Range
1	Anti-Neurofascin 155 (NF155) Antibody	Cell-Based Assay (CBA)	Negative (–)	Negative
2	Anti-Neurofascin 186 (NF186) Antibody	Cell-Based Assay (CBA)	Negative (–)	Negative
3	Anti-Contactin-1 (CNTN1) Antibody	Cell-Based Assay (CBA)	Negative (–)	Negative
4	Anti-Contactin-2 (CNTN2) Antibody	Cell-Based Assay (CBA)	Negative (–)	Negative
5	Anti-CASPR1 (Contactin-associated protein-like 1) Antibody	Cell-Based Assay (CBA)	Negative (–)	Negative
**Serum Myelin-Associated Glycoprotein (anti-MAG) antibody testing**				
1	(anti-MAG) Myelin-Associated	ELISA	Negative (–)	Negative

	Glycoprotein antibody			
**Autoantibody Profile for Connective Tissue Disease (ANA Panel, 19 Items)**				
No.	Autoantibody Name	Method	Result	Reference Range
1	Antinuclear Antibody (Hep-2 Cell, Nuclear Pattern)	Immunofluorescence	Negative (–)	Negative
2	ANA Titer	Immunofluorescence	Negative (–)	Negative
3	Antinuclear Antibody (Hep-2 Cell, Nuclear Pattern)	Immunofluorescence	Positive	Negative
4	ANA Titer	Immunofluorescence	Negative (–)	Negative
5	Anti-dsDNA Antibody	Immunoblot	Negative (–)	Negative
6	Anti-nRNP/Sm Antibody	Immunoblot	Negative (–)	Negative
7	Anti-Sm Antibody	Immunoblot	Negative (–)	Negative
8	Anti-SSA Antibody	Immunoblot	Negative (–)	Negative
9	Ro-52 Antibody	Immunoblot	Negative (–)	Negative
10	Anti-SSB Antibody	Immunoblot	Negative (–)	Negative
11	Anti-Scl-70 Antibody	Immunoblot	Negative (–)	Negative
12	Anti-PM-Scl Antibody	Immunoblot	Negative (–)	Negative
13	Anti-Jo-1 Antibody	Immunoblot	Negative (–)	Negative
14	Anti-Centromere Protein B Antibody	Immunoblot	Negative (–)	Negative
15	Anti-Proliferating Cell Nuclear Antigen Antibody	Immunoblot	Negative (–)	Negative
16	Anti-Nucleosome Antibody	Immunoblot	Negative (–)	Negative
17	Anti-Histone Antibody	Immunoblot	Negative (–)	Negative
18	Anti-Ribosomal P Protein Antibody	Immunoblot	Negative (–)	Negative
19	Anti-Mitochondrial Antibody M2	Immunoblot	Negative (–)	Negative
**ANCA and Glomerular Basement Membrane Antibody Profile (12 Items)**				
No.	Autoantibody Name	Method	Result	Reference Range
1	Cytoplasmic ANCA (cANCA, 1:10)	Indirect Immunofluorescence	Negative (–)	Negative

2	Cytoplasmic ANCA (cANCA, 1:32)	Indirect Immunofluorescence	Negative (–)	Negative
3	Cytoplasmic ANCA (cANCA, 1:100)	Indirect Immunofluorescence	Negative (–)	Negative
4	Perinuclear ANCA (pANCA, 1:10)	Indirect Immunofluorescence	Negative (–)	Negative
5	Perinuclear ANCA (pANCA, 1:32)	Indirect Immunofluorescence	Negative (–)	Negative
6	Perinuclear ANCA (pANCA, 1:100)	Indirect Immunofluorescence	Negative (–)	Negative
7	Antinuclear Antibody (ANA, IIF 1:25)	Indirect Immunofluorescence	Negative (–)	Negative
8	Anti-GBM Antibody (1:10)	Indirect Immunofluorescence	Negative (–)	Negative
9	Anti-GBM Antibody (1:32)	Indirect Immunofluorescence	Negative (–)	Negative
10	Anti-GBM Antibody (1:100)	Indirect Immunofluorescence	Negative (–)	Negative
11	Anti-Myeloperoxidase Antibody (Anti-MPO)	Immunoblot	Negative (–)	Negative
12	Anti-Proteinase 3 Antibody (Anti-PR3)	Immunoblot	Negative (–)	Negative

Peripheral Blood T Lymphocyte Subset Analysis

No.	Marker	Parameter Description	Result	Unit	Reference Range
1	CD3+%	Total T lymphocyte percentage	57.22	%	50–84
2	CD3+	CD3+ T cells	886.00	cells/µL	876.8–2310
3	CD4+%	T helper cell percentage	31.64	%	27–51
4	CD4+	CD4+ T helper cells	489.40	cells/µL	455.7–1261
5	CD8+%	Cytotoxic T cell percentage	27.90	%	15–44
6	CD8+	CD8+ cytotoxic T cells	431.50	cells/µL	238–1250
7	CD4/CD8	CD4/CD8 ratio	1.13	—	0.71–2.78

Table 4. Summary of cerebrospinal fluid (CSF) studies at two time points

Day (rel	Opening	Protei	Red blo	Nucleate	Glucose	IgG	IgM	IgA	OCB	CNS autoi	mNGS	Interpretation	India	Cryptococc	Gram	Acid-	CSF	PCR for

ative)	pressure (mmH$_2$O)	n (g/L)	od cells (×10^6/L)	d cells (×10^6/L)	(mmol/L)	(mg/L)	(mg/L)	(mg/L)		mmune antibody panel		(key points)	Ink Stain	al Antigen	Stain	Fast Stain	Culture	Mycobacterium tuberculosis
Day 7	>300	0.51	86	2	4.65	38.91	0.66	6.62	Negative†	Negative	Negative	Albumin cytologic dissociation (ACD)	Negative	Negative	Negative	Negative	Negative	Negative
Day 16	220	0.67	21	1	6.48	61.63	2.38	21.45	Negative†	Negative		persistent ACD	Negative	Negative	Negative	Negative	Negative	

Table5. Peripheral Nerve Conduction Study Results

Nerve & Muscle	Min-F (ms)	Max-F	Dispersion	Persietence	Mean-F	L-R Mean-F	F/M Ratio	F-M Lat （ms）
**Left Ulnar Nerve (Abductor Digiti Minimi)**								
	28.36	30.78	2.42	100.00	29.331		1.33	25.94
**Left Common Peroneal Nerve (Extensor Digitorum Brevis)**								
	56.88	59.53	2.65	50.00	58.28		0.97	55.78
**Left Tibial Nerve (Abductor Hallucis)**								
	58.28	62.66	4.38	100.00	60.91	5.14	3.60	
**Right Tibial Nerve (Abductor Hallucis)**								
	53.13	58.13	5.00	100.00	55.77	5.14		
**Left Median Nerve (Abductor Pollicis Brevis)**								
	27.73	30.23	2.50	80.00	29.05	1.12	24.38	

Note: Prolonged F-wave latencies in the ulnar, common peroneal, median, and tibial nerves indicate peripheral demyelination.

Table6. Somatosensory/Visual Evoked Potential Reports

Somatosensory Evoked Potentials (Lower Limb Stimulation)	Latency (ms)	Latency (ms)	Latency (ms)	Latency (ms)	Latency (ms)	Latency (ms)	Peak-to-Peak(μV)
	P40	N50	P60	N8	N8	P40 P60	N50
**Right 19.5mA/Cz**	41.3	55.0	69.3		10.8	28.0	0.31
**Right 19.5mA/Cz**	39.8	53.5		8.83		27.0	0.66
**Right 19.5mA/Popliteal Fossa**			66.8		34.3		
**Left**	42.0	61.3	71.3			29.3	1.11

**22.5mA/ Cz**							
**Left 22.5mA/ Cz**	42.3	59.7	78.8			36.5	1.13
**Left 22.5mA/Popliteal Fossa**				8.83	11.0		
**Visual Evoked Potentials (Pattern-Reversal Stimulus/Recording)**	Latency (ms)	Latency (ms)	Latency (ms)	Peak (ms)	Peak-to-Peak(μV)	Peak-to-Peak(μV)	
	N75	P100	N145	N75N145	N75P100	N145P100	
**Right (Full-field 16x12/0z)**	71.0	103.5	141.5	50.5	0.24	7.67	
**Left (Full-field 16x12/0z)**	72.0	108	164.0	92.0	6.60	8.75	
**Visual Evoked Potentials (LED Flash Stimulus/Recording)**	Latency (ms)	Latency (ms)	Latency (ms)	Peak (ms)	Peak-to-Peak(μV)	Peak-to-Peak(μV)	
	Na	P100	Nb	Na Nb	Na P100	NbP100	
**Right 10ms/0z**	55.0	105.5	162.5	107.5	14.0	15.2	
**Right 10ms/0z**	67.0	115.0	196.5	129.5	7.74	3.80	
**Right 10ms/0z**	73.0	126.5	175.0	102.0	2.14	3.13	
**Left 10ms/0z**	68.0	122.0	155.0	87.0	4.49	1.30	
**Left 10ms/0z**	65.5	120.0	180.0	114.5	1.28	3.79	
**Left 10ms/0z**	55.0	122.0	162.0	107.0	4.46	1.55	
**Visual Evoked Potentials (Left Hemifield Stimulus/Recording)**	Latency (ms)	Latency (ms)	Latency (ms)	Peak (ms)	Peak-to-Peak(μV)	Peak-to-Peak(μV)	
	N75	P100	N145	N75N145	N75P100	N145P100	
**Right-left (16x12)**	66.0	130.0	125.0	59.0	66.3	1.37	
**Left-left (16x12)**	55.5	97.0	172.0	116.5	4.28	7.78	

Note: Somatosensory evoked potentials (SEPs) of the lower extremities demonstrated bilaterally diminished cortical differentiation accompanied by markedly reduced amplitudes. While the P40 latencies were observed at the upper limit of the normal range, bilateral N8 components remained within normative values. Visual evoked potential (VEP) testing revealed distinct abnormalities: Left eye stimulation (full-field and left hemifield) produced poorly differentiated cortical waveforms with decreased P100 amplitudes, alongside prolonged P100 latency in full-field testing. Right eye full-field stimulation similarly exhibited disorganized cortical potentials with a significant reduction in P100 amplitude. These electrophysiological patterns collectively indicate central nervous system pathology affecting both the proprioceptive pathways of the lower extremities and the visual processing pathways.

Table 7. Initial Flow Cytometry Results (B-cell Subsets)

Parameter	Method	Result	Unit	Flag	Reference Range
CD19+ B cells / lymphocytes	Flow Cytometry	43.51	%	↑	3.80–21.50
CD19+ B cells absolute	Flow Cytometry	273.37	cells/μL		51.00–728.00
Memory B cells % (CD19+CD27+CD38dim)	Flow Cytometry	15.32	%		11.00–46.60
Memory B cells absolute	Flow Cytometry	41.88	cells/μL		3.00–80.00
Switched Memory B cells %	Flow Cytrometry	13.95	%		4.60–35.50
Switched Memory B cells absolute	Flow Cytometry	38.14	cells/μL		1.00–53.00
Plasmablasts %	Flow Cytometry	3.90	%		0.30–7.80
Plasmablasts absolute	Flow Cytometry	11.87	cells/μL	↑	0.00–6.00
Memory B cells % of mononuclear cells	Flow Cytometry	4.18	%		

Table8. Follow-up Flow Cytometry After Rituximab Treatment

Parameter	Method	Result	Unit	Flag	Reference Range
CD19+ B cells / lymphocytes	Flow Cytometry	0.03	%	↓	3.80–21.50
CD19+ B cells absolute	Flow Cytometry	0.25	cells/μL	↓	51.00–728.00
Memory B cells % (CD19+CD27+CD38dim)	Flow Cytometry	66.67	%	↑	11.00–46.60
Memory B cells absolute	Flow Cytometry	0.17	cells/μL	↓	3.00–80.00
Switched Memory B cells %	Flow Cytrometry	66.67	%	↑	4.60–35.50
Switched Memory B cells absolute	Flow Cytometry	0.17	cells/μL	↓	1.00–53.00

Plasmablasts %	Flow Cytometry	0.00	%	↓	0.30–7.80
Plasmablasts absolute	Flow Cytometry	0.00	cells/µL	↑	0.00–6.00
Memory B cells % of mononuclear cells	Flow Cytometry	0.01	%		

2 Supplementary Figures and Tables

2.1 Supplementary Figures

Figure 1. Diagnostic and therapeutic timeline of a patient with combined central and peripheral demyelination.

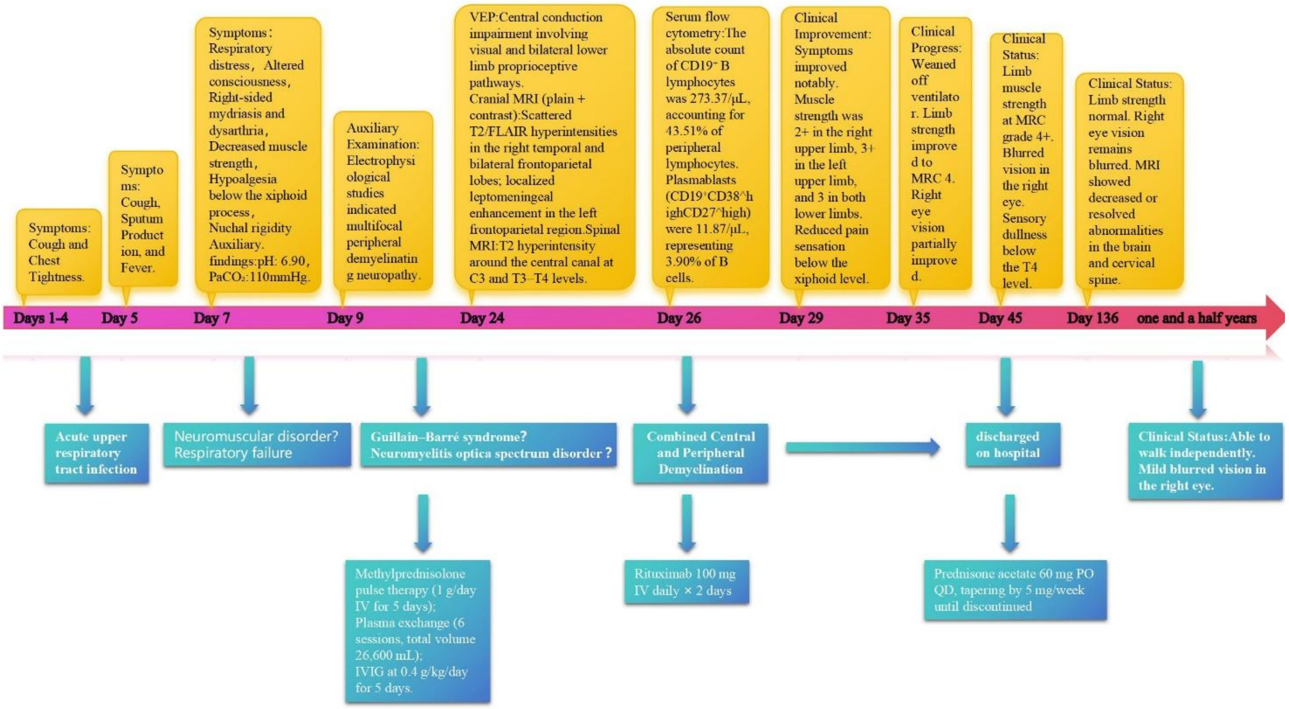

Figure 2. Longitudinal IL 6 and B cell (plasmablast) dynamics aligned with therapy milestones and ventilatory course. Detailed data are provided in Supplementary Table 1.

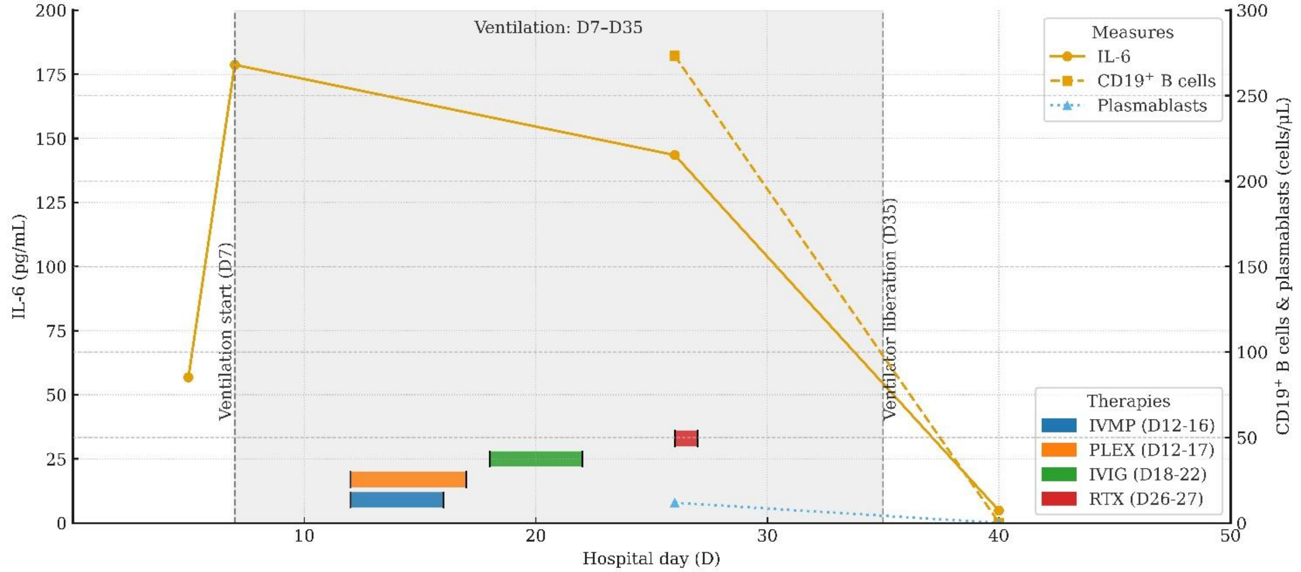

Figure 3. Neuroimaging findings and follow-up MRI

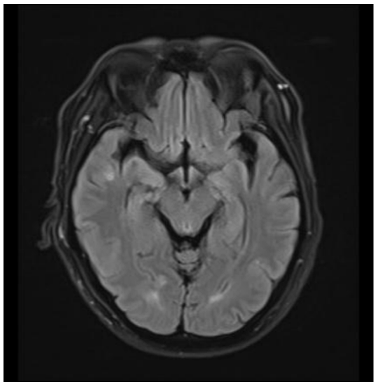
Figure 3a

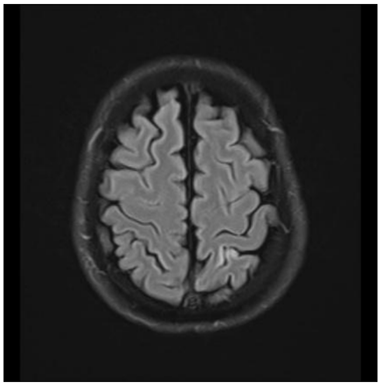
Figure 3b

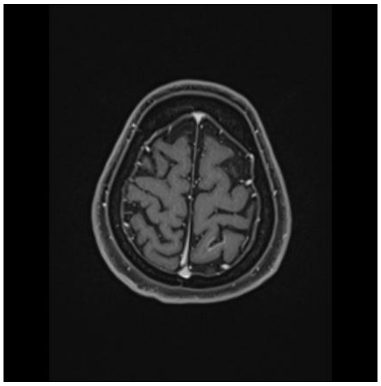
Figure 3c

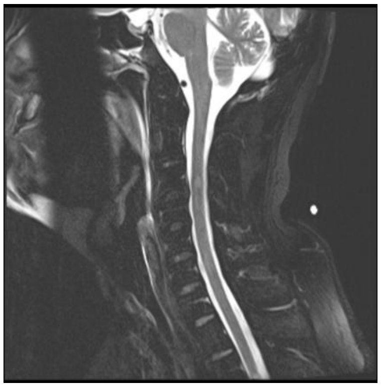
Figure 3d

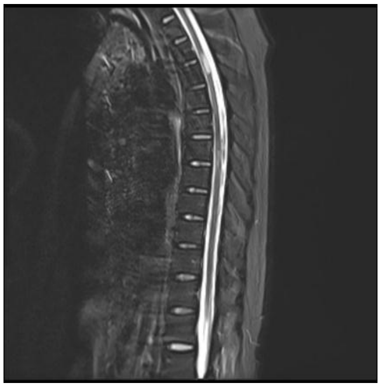
Figure 3e

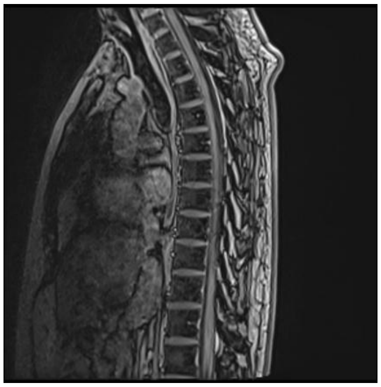
Figure 3f

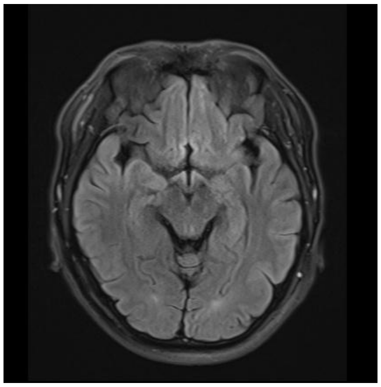

Figure 3g

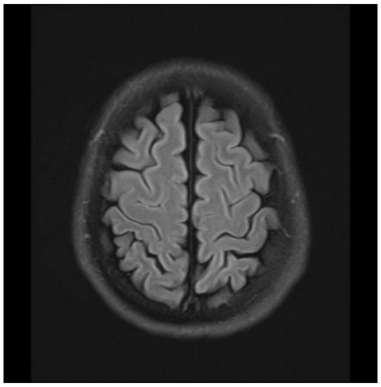

Figure 3h

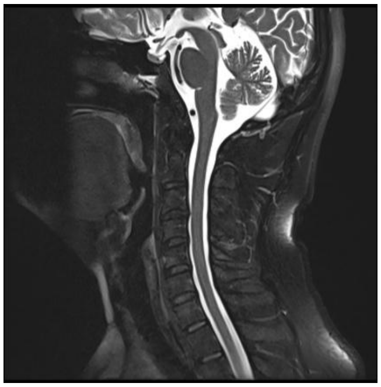

Figure 3i

(3a) Scattered punctate T2-weighted and FLAIR hyperintense lesions in the right temporal and frontoparietal lobes. (3b) Similar scattered hyperintense lesions in the left frontoparietal cortex on T2-weighted and FLAIR sequences. (3c) Post-contrast T1-weighted imaging shows focal leptomeningeal enhancement in the left frontoparietal region. (3d) T2 hyperintensity surrounding the central canal at the C3 level of the cervical spinal cord. (3e) T2 hyperintensity around the central canal at the T3–T4 levels of the thoracic spinal cord. (3f) Mild contrast enhancement of the T3–T4 spinal cord lesions on post-contrast T1-weighted images. (3g) Follow-up T2-FLAIR images demonstrate resolution of the hyperintense lesions in the right temporal and frontoparietal lobes. (3h) Decreased FLAIR hyperintensity in the left parietal lobe on follow-up imaging. (3i) Resolution of previously abnormal T2 signal in the cervical spinal cord.

Figure 4. Scatter Plot of B Cell Analysis by Flow Cytometry

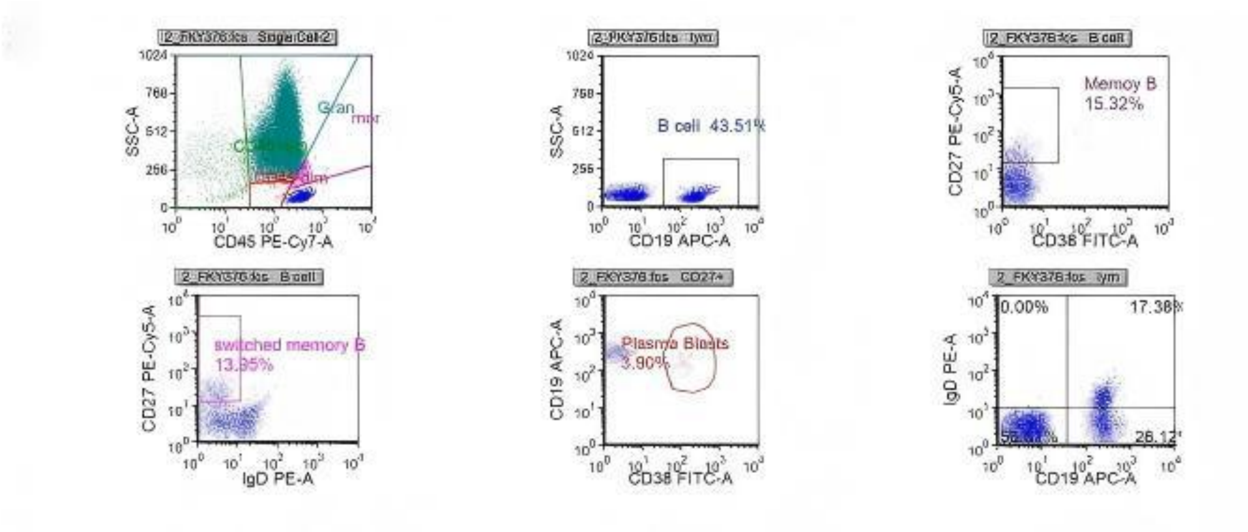

(4a) B-cell population ($CD19^{+}$) on day 26.

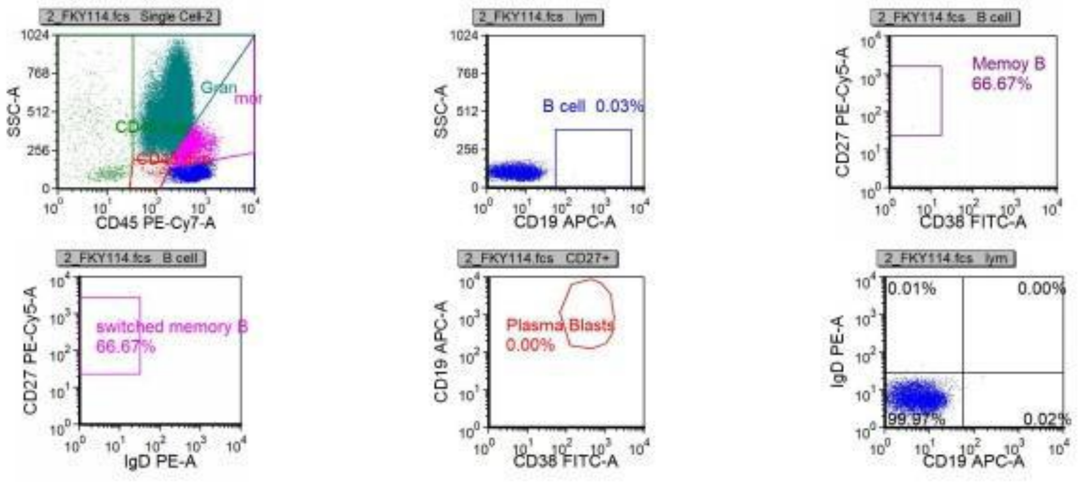

(4b) B-cell population ($CD19^{+}$) on day 40.
